# Smart medical beds in patient-care environments of the twenty-first century: a state-of-art survey

**DOI:** 10.1186/s12911-018-0643-5

**Published:** 2018-07-09

**Authors:** Ignacio Ghersi, Mario Mariño, Mónica Teresita Miralles

**Affiliations:** 1 Centro de Investigación en Diseño Industrial de Productos Complejos (CIDI-FADU-UBA), Facultad de Arquitectura, Diseño y Urbanismo, Universidad de Buenos Aires, 2160 Intendente Güiraldes Ave, Buenos Aires, Argentina; 2 Laboratorio de Biomecánica e Ingeniería para la Salud (LaBIS-FI-UCA), Facultad de Ingeniería y Ciencias Agrarias, Pontificia Universidad Católica Argentina, 1600 Alicia Moreau de Justo Ave, Buenos Aires, Argentina

**Keywords:** Electric bed, Smart medical bed, Patient-centered, Technology, Accessibility

## Abstract

**Background:**

Recent scientific achievements and technological advances have brought forward a massive display of new or updated medical devices, enabled with highly-developed embedded-control functions and interactivity. From the final decade of the twentieth century, medical beds have particularly been affected by this surge, taking on new forms and functions, while accommodating to established properties that have become well-known for these devices. The past fifteen years have also brought forward changes to conceptual frameworks, concerning the product design and manufacturing processes (standards), as well as the patient (perspectives on patient-care environments and accessibility). This work presents a state-of-art survey on electric medical beds, representing what is defined as the time of “smart beds”, as part of an increasingly comprehensive patient-care environment.

**Methods:**

A survey and assessment of market trends, research efforts and standards related to smart medical beds was performed, covering a wide range of public records of intellectual property, models and related healthcare solutions, as well as relevant research efforts in the field between 2000 and 2016. Contextual topics, necessary for the understanding of this subject, on novel technologies, disability and the reach of healthcare systems, were also researched and interpreted.

**Results:**

The new generation of electric medical beds is defined, with the final stage of the proposed timeline for these devices being covered. Functional, aesthetic and interactive features are presented, and the current global market for medical beds and related standards are also assessed. Finally, discussions concerning rising challenges and opportunities for these systems are explored, with the potential for adding further monitoring and assistive implementations into medical devices and environments being highlighted.

**Conclusions:**

Smart medical beds are integrated solutions for patient care, assistance and monitoring, based on a comprehensive, multidisciplinary design approach. Research in this field is critical in a context of global ageing, and powered by a surge in opportunities for accessibility solutions. Smart beds, seamlessly integrated into the healthcare system, have a unique opportunity in enabling more efficient efforts for caregivers, and more responsive environments for patients.

## Background

Electric medical beds have accumulated almost one hundred years of history. An essential part of the healthcare environment, the medical bed is also used as a measure of its *reach* [1], its *efficiency* (for occupancy and bed-management strategisation [2]), *development* (representing funding and investment in healthcare systems, see [3]) and *diversity*. For the case of automated, electric devices such as these, technological and contextual factors have resulted in significant changes to their appearance and their expected functionality over this period, while retaining original features that have guided the first exponents of this medical device. It is, however, in the twenty-first century, that an unprecedented, innovative stage in the development of these devices has peaked, taking advantage of all technological means at the disposal of developers, and resulting in new vectors of added value for these products: this stage can be referred to as the time of *smart medical beds*.

Previous work [4] has detailed the evolution of medical beds form initial, push-button models, to the year 2000, dividing such period into two stages: *electric beds* (1940’s to 1980’s) and *mechatronic*^1^ *beds (1990’s)*. Highlights from this diachronic study over a sixty-year period were:

- Spreading of these devices outside the hospital environment from the 60’s, towards institutional or residential facilities.
- Development of particular regulatory frameworks
- Growing invention and commercialization of dedicated accessories
- Incipient incorporation of new technologies into the devices (second stage)

Mechatronic beds became a reality in the 90’s, when inventions, commercial products (i.e. Hill-Rom TotalCare-1998), and dedicated research work [5] accumulated ergonomics, functions and accessories (alternative actuators, pressure mattresses, weighing scales), incorporating informatics and communications into these devices.

In the past decades, the medical-bed market has further changed, responding to also-changing structural, functional, and social-economic demands concerning the performance of medical beds. From the year 2000 to the present, these highly elaborate mechatronic devices have consolidated into what can be called the segment of smart mechatronic beds or *smart beds*, a term that describes a comprehensive *synthesis* between new materials, design and higher functionality and autonomy for these systems, all under advanced user interfaces. Smart beds implement new technologies (graphical interfaces, novel environment-aware sensors and actuating solutions, etc.), to provide a higher level of service and function, like real-time monitoring, caregiver and patient assistance, automated functions and positions (chair, assisted bed exit), and data logging, as well as more advanced means of communication.

This work presents a survey on medical beds in the healthcare environments between the years 2000 and 2016, leading to an analysis on their current product and research state-of-art, as well as on their potential for development and market perspectives. Focused on the final stage of the product timeline, it proposes a study of wide scope on the many factors affecting medical beds over this reduced time range, through a deeper assessment of current technologies, challenges and views that are set to shape the healthcare environment and delivery methods in the near future.

## Methods

This work presents the results of a survey on electric medical beds from the year 2000, and up to 2016. Three different topics were assessed:

- Trends and changes found in medical beds over this period (section “Twenty-first century medical beds: Trends and changes (2000–2016)”).
- Current market-reach and features of smart beds (section “Twenty-first century medical beds: Current market-reach and features”).
- Research efforts with an impact on the experience or capabilities of the medical bed, as part of more comprehensive healthcare environments (section “Research shaping the future of smart healthcare environments”).

As a characterization of the current state of a specific medical device, sources for this article have been the result of a thorough literature survey on its core subject (medical, long-term care beds, patients and operators). In order to form a comprehensive view on the matter and its perspectives, however, other relevant sources representing economic, social/political and cultural implications to such a relevant medical device were also involved in this study:

- Current models of medical beds (significant brands, models of medical beds, and products found through dedicated catalogs [6]).
- Relevant research findings and publications (*IEEExplore, JSTOR, PubMed, SpringerLink, EBSCOhost, ScienceDirect*), concerning mechatronics, patient/bed monitoring, advances in user interfaces, accessibility and sensors. Terms (with combinations): *Hospital Bed/s, Control, Bedridden, Stretcher, MEMS, Face* (for face control in accessibility*), BCI, Ergonomics, Motor Impairment, South America* (for local research and product state-of-art)*, Trends, Long term, Technology, Cost Nursing Beds, Design.*
- Public records of intellectual property (hospital/electric bed control interfaces, accessibility-enabled beds, networking and communication): United States Patent and Trademark Office, German Patent and Trademark Office, The Lens database, INPI (Argentina).
- Standards relevant to the implementation of new technologies into these devices, based on reports from regulatory agencies and certifications declared by medical-bed models.
- Commercial solutions and technological news at a global scale on these topics (*IEEE Spectrum, Scientific American, Wired Magazine*, *Popular Mechanics).* Terms: *BCI, Bedridden, Hospital, Bed, Control, Face Control, MEMS*.

Inclusion criteria restricted models between 2000 and 2016, and to those designed for medium to long-term care scenarios, for which patient/caregiver/environment interactions become most relevant. Scientific publications included research on novel sensors and assistive technologies, implemented or believed to be compatible with the use or improvement of the experience of the medical bed (motion sensors, brain-computer interfaces, image processing, speech recognition, and adapted push-buttons). Changes to regulations covering medical beds in this period were also assessed. Unlike commercial models of medical beds, patents were not restricted to the period 2000–2016 because the priority or issue date may not be indicative of when the invention is actually implemented or distributed. Criteria for the evaluation of innovations and changes to medical beds in this period were a) user interfaces, b) accessories, c) aesthetic-morphological properties, and d) embedded functions.

## Results

A total 85 distributors and manufacturers of medical beds from America, Europe and Asia were found and evaluated. A wide range of beds covering home-care, institutional-care, emergency, surgical and therapeutic beds (bariatric, elderly, long-term) have been detected. Given the inclusion criteria of this work, emergency, psychiatric, obstetric and surgical beds were not assessed. Nine companies were found to be distributors from other brands. Out of the rest, two manufacturers were discarded for lack of data concerning the specifics of the manufactured beds, resulting in a group of 74 manufacturers of medical beds.

**Fig. 1:**
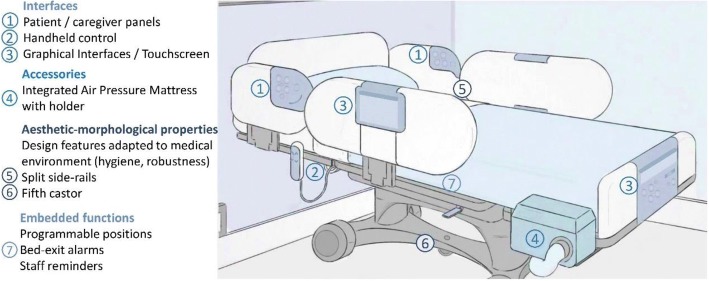
Illustration of a smart medical bed for clinical use: directed at multiple settings, smart medical beds integrate an array of innovative interfaces, functions and accessories, with distinct design features and customizations

Figure 1 is an illustration of a smart medical bed summarizing the changes that were found to be most significant to these medical devices in the twenty-first century: innovative interfaces, increased functionality and dedicated accessories, with customization and finishing options.

Integration of features like those in Fig. 1 into medical beds has been a result of technological advances of the past decades. Table 1 is a list of records of intellectual property, found to be associated to smart features compatible with medical beds and patient-care environments of this period (see Section “Smart features”). Accessibility, networking and environmental control from the bed, as well as improved function and design features are exemplified in these patents, with many of these concepts or variations currently available in smart beds.

**Table 1:** Selected records of intellectual property concerning smart functions, design features and connected patient-care environments

Priority Year	Ref	Patent	Title	Highlight
1991	[35]	US5335313A	Voice-actuated (…) control system for hospital bed	Speech Recognition
1992	[17]	US6481688	Hospital bed communication and control device	Accessibility Accessory for Beds
1994	[48]	US5831221A	Caster mounted weighing system	Integrated Scale
	[49]	US5454126A	Foot egress chair bed	Chair Position
1995	[50]	US5542138	Bedside control unit for a hospital bed	Environmental Control through bed Adjustable interface
	[51]	US7017208B2	Hospital Bed	Graphical Interface
1998	[52]	US6781517B2	Communication and Bed Function Control Apparatus	Multiple Integrated Controls
2001	[53]	US8334779	Touch Screen Control of a Hospital Bed	Touchscreen
2004	[8]	US7852208B2	Wireless Bed Connectivity	Networking Functionality
2005	[10]	US20080172789A1	Patient Support with Improved Control	Integrated Front Panel, Side-rail Controls (patient and caregiver). Touchscreen, Graphical Interface. Environmental Control
2007	[54]	EP2027844A1	Proximity Activation of Voice Operation of Hospital Bed	Different Operation for Caregiver and Patient, Voice control
2010	[11]	US20110214234A1	Multifunctional Display for Hospital Bed	Touchscreen for Patient and Caregiver. Environmental control
2010	[55]	EP2438897A2	Hospital bed with graphical user interface having advanced functionality	Graphical User Interface, Networking, Communication
2010	[56]	EP2460503A2	Biometric Bed Configuration	Biometrics, Conditional Control

Table 2 lists 20 models from 13 manufacturers that have been selected as representative of the different criteria that were chosen for the evaluation of smart beds (user-interfaces, accessories, aesthetics/design, functions). Highlights of these models are further explored in sections “Twenty-first century medical beds: Trends and changes (2000–2016), Twenty-first century medical beds: Current market-reach and features and Research shaping the future of smart healthcare environments”, with each section detailing findings on the three areas of interest that were listed in section “Methods”.

**Table 2:** Smart medical beds: models exhibiting state-of-art functional, design and interactive features

Brand	Model	Year (where available)
ArjoHuntleigh	Enterprise 9000	2010
Haelvoet	Olympia Hospital	2013
Hill-Rom	TotalCare SpO_2_RT	2004
	TotalCare Bariatric	2007
	Excel Care ES	2013
Joerns	Bari10A	2012
	UltraCare XT	2013
Linet	Eleganza Smart	2009
	Eleganza 3XC	2013
Merivaara	Carena	2012
Paramount Bed Co. Ltd.	Rakusho Series	–
	METIS VIP Series	–
	KA6600 Series	–
Pro Bed	Freedom Bed	–
Rotec	Versatech 600	2013
SizeWise	Navigator	2013
Stryker	InTouch II	2008/2011
	S3 Med/Surg Bed	2015
Vallitech	VLT-931	2015
Völker	LTC Beds	2016

### Twenty-first century medical beds: Trends and changes (2000–2016)

#### Market expansion and technological availability

In accordance with the aforementioned results, a growing number and variety of medical beds has been found in this period, with increased mechanical and autonomous functions, reaching up to novel devices, like a continuous-use bed that turns into a wheelchair for disabled patients [7], and models with embedded networking, communication, monitoring and integrated alarms [8] (i.e. Stryker iBed Awareness). The global market for medical beds has become, as a result, vastly more competitive in the past decades [9], and the continuing trend of specialization into different sub-groups of patients and environments is evident: apart from *bariatric* (i.e. Joerns Bari10A and Hill-Rom Excel Care ES) and *domiciliary* beds, new product lines, aimed at the aid of the *elderly* (Japan’s Paramount Bed Co. Ltd. Rakusho Series) or *pediatric* patients appear more prominently, while other models (for instance Merivaara Carena) display features directed at many of these populations under a single model.

Following the trend of all medical devices, the integration of more-developed technologies into electric medical beds became the most significant in this period, resulting in examples with multi-language speech synthesis for patient-caregiver communication (Stryker InTouch), and recommending [10], and even implementing speech-recognition functions for their control (Vallitech-2015). With significant embedded capabilities and autonomy, user interfaces (detailed in section “User interfaces”) continue to stand out as relevant weighting factors in the evaluation of these products. A patent of 2005 shows multiple potential configurations for side-rail integrated controls [10], and a subsequent patent of 2010 updates these alternatives with touchscreen functions, for both patient and caregiver use [11]. In 2004, a patent displaying a bed with wireless connectivity, high-complexity user interfaces, and incorporating patient-blocking functions, foot pedals, and automated functions was presented [8]. The TotalCare SpO_2_RT bed, from Hill-Rom, as well as Stryker’s InTouch Care bed and SizeWise’s Navigator models, show integrated touchscreens and dedicated graphical user interfaces as part of their developments. Upgraded, comprehensive front panels and alternate controls continued to proliferate in this period, and the redundancy of controls became a standard feature (for instance, visible in Haelvoet Olympia Hospital and Linet Eleganza Smart models). Improved ergonomics and risk-reducing articulations have also been included into new devices, modifying the hip-section articulation when the backrest is lifted (TotalCare SpO_2_RT model), and/or compensating the position of the patient in its environment (Stryker S3 Med/Surg Bed).

#### Changing (and keeping) the face of the medical bed

Over the past years, trends and most relevant innovations to medical beds have been notably related to design aspects, referred to materials (more hygienic and resistant), population-specific models, ergonomic manual commands, and morphological changes associated to updated mobility-options (elevation, front-back and lateral inclination, etc.), updated side-rails, patient-support structures, castors (fifth castor for enhanced transport), while embedded with the aforementioned new technologies.

In the competitive scenario presented in previous sections, aesthetic and comprehensive design features stand out as a differentiating factor between products. These features, prominently represented by the side-rails and panels, also serve in adapting the beds to different environments and populations. An analysis of these products, and their means of diffusion, allowed inferring a set of values and responses that this new generation of smart beds aims to provoke:

- They are highly and increasingly user-oriented (institutions, operators and patients), which is expressed in their form, function, interactivity and ergonomics.
- They strongly express the ideas of hygiene, safety, durability, comfort, ease of use (both for patients and caregivers), reliance on technology and caregivers, with panels and side-rails as main differentiating factors (full/split rails, integrated controls).
- They emphasize embedded functions and high-end technologies, displaying the power embedded into these products, and in line with a growing technological awareness. However, they also allude to the invisibility of this elaborate technology with simple, intuitive controls.

**Fig. 2:**
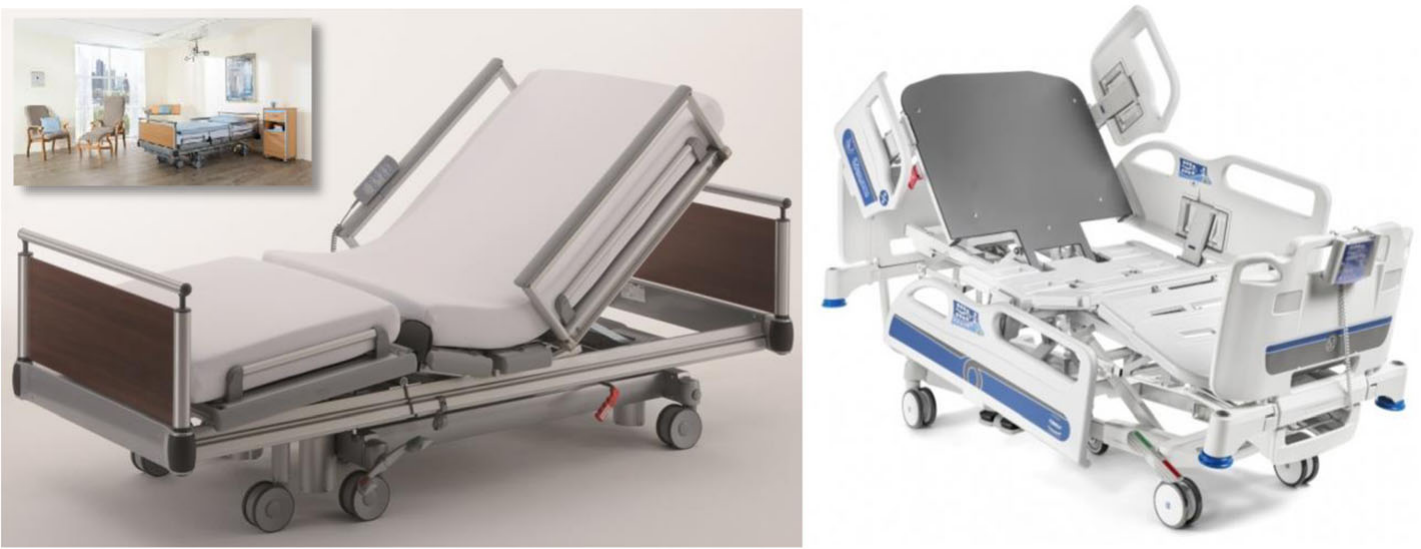
Aesthetic and design customizations stand out in state-of-art medical beds, serving a purpose of adaptation to different environments. Left: residential-inspired, long-term care (Völker LTC Vis-à-vis bed) [43]; Right: cardiac and progressive-care for hospital ward (Malvestio Sigma PCU Electric Bed Scale System) [44]. Permission for use of images granted by Völker and Malvestio

In sum, the following trends, exemplified in Fig. 2, have been associated to the design of smart beds:

- While these devices highlight their advanced functionality, they maintain simplified control interfaces, relying on known structures and signals, to ease their use by patients or caregivers.
- For *residential* or *residential-inspired beds*, they assimilate into these daily environments (Haelvoet Olympia Hospital, Völker LTC Beds, Paramount Bed Co. Ltd. METIS VIP).
- Oppositely, *critical-care beds* highlight robustness, embedded technologies, and their reliability for medical use through their design (Stryker InTouch, Hill-Rom TotalCare Bariatric, Linet Eleganza 3XC, Paramount Bed Co. Ltd. KA6600 series). Other models can be customized for both scenarios (i.e. Rotec Versatech 600).

#### Regulatory consolidation

Regulatory frameworks have an impact on the design-process and requisites expected of different families of devices in order to reach their potential users. In the sector of medical beds, there have been advances in this field between 2000 and 2016. After the development of particular standards for electric medical beds in the late 90’s [4], the first decades of the twenty-first century have seen the convergence of these standards into a unified reference. ISO/IEC standard 60,601–2-52:2009 (basic safety and essential performance of medical beds) [12] was issued in 2009 as a combination of previous standards IEC 60601–2-38:1996 (requirements for the safety of electrically operated hospital beds) [13], and EN 1970:2000 (adjustable beds for disabled persons: requirements and test methods). This document, which covers issues like safe working loads, mechanical and electrical safety, ergonomic requisites and risk-management strategies as a consolidated reference among five scenarios for medical beds (from hospital to nursing and home environments) [12], was first amended in 2015 [14], and falls within the updated scope of IEC 60601 [15].

### Twenty-first century medical beds: Current market-reach and features

#### Current market reach

The current number of developers and manufacturers of medical beds, their associated products and accessories, as well as healthcare-management technologies, falls easily within the hundreds. Significant market actors, as described by medical-bed market surveys [9, 16], include Hill-Rom, Linet, ArjoHuntleigh, Stryker, Paramount Bed Co. Ltd. and Invacare.

**Fig. 3:**
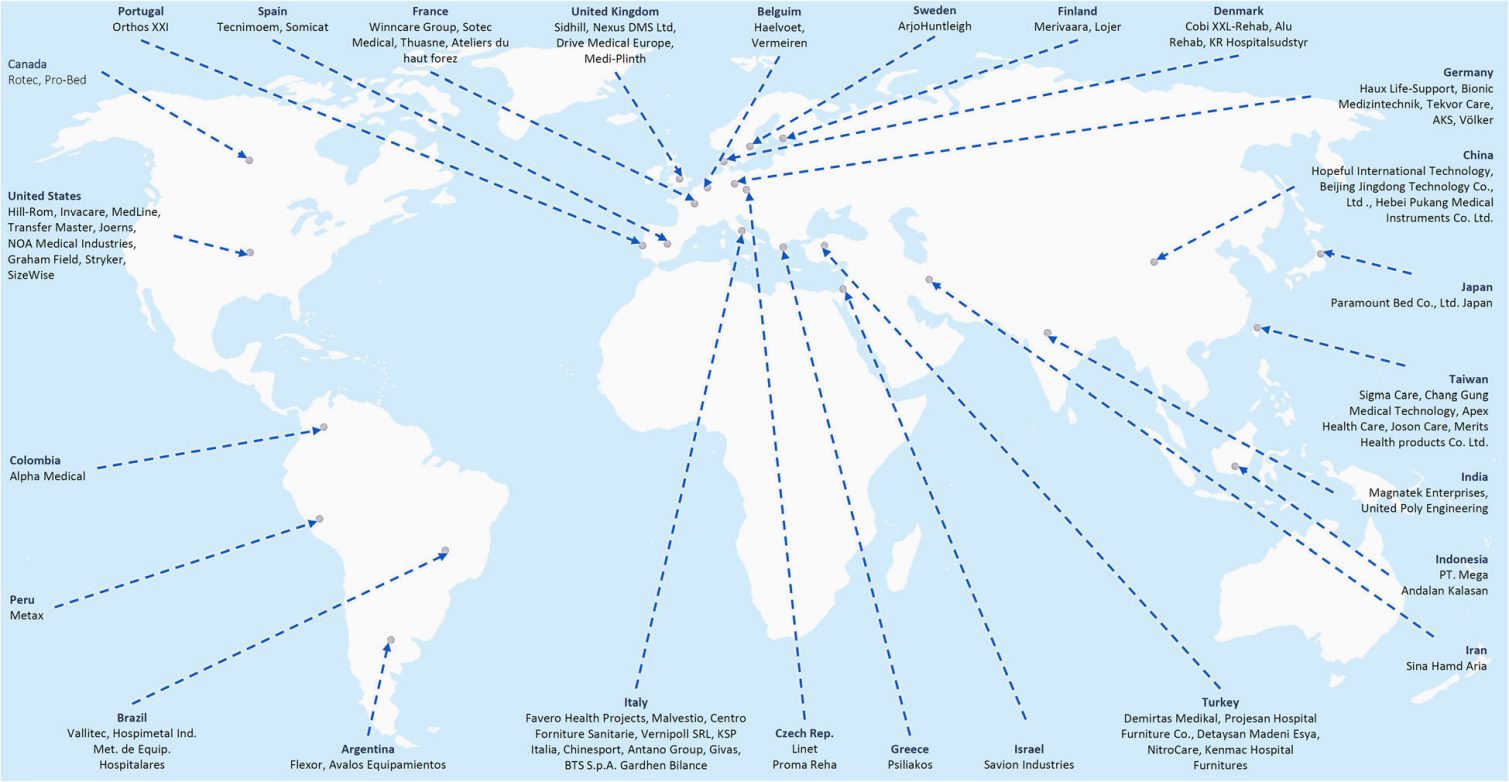
Global distribution of the reviewed electric and smart-bed manufacturers. World map adapted from [45]

The global market of electric and smart medical beds, both for healthcare facilities and residential use, reaches its highest degree of development in the United States and Europe, with the Asian market showing great potential for growth in the following years, and within this market, pressure-relief surfaces and beds are among the most prominent sub-groups [9]. Figure 3 shows the global distribution of the reviewed companies (accessed 04/2018).

#### Smart features

Table 3 shows a set of stand-out curent medical-bed properties found on the reviewed systems, ranging from homecare to intensive-care solutions, detailing *aesthetic-morphological properties* (section “Changing (and keeping) the face of the medical bed”) and *special functions* that are available in these devices, stemming from the integration of multiple technological solutions into one device (section “Market expansion and technological availability”). As a group, these devices directly highlight the different *specific purposes* that medical beds can be intended for (see “description”), as well as the increased level of technology embedded into them over time. These features are divided in the table, according to their relation to the *device* itself, the *user* (caregiver or patient), or the *healthcare environment*.

**Table 3:** Standout features of current medical beds

	Related to the device	Related to the patient/caregiver	Related to the environment
Description	• Ergonomic • Low: controllable frame height, significantly lowering the frame to mitigate consequences of bed falls	• Universal • Bariatric • Critical • Acute • Early Mobility	• Hospital • Intensive Care • Long-Term • Residential • Medical-Surgical • Multiple
Aesthetic/design features	• 5th castor for eased bed transportation • Robustness, removable panels for hygiene • Controllable height (including low-frame models) • Side-rail shock absorbers • Advanced mechanics	• Frame extension (length-width) • Ergonomics for patient, caregiver • Movable, adaptable side-rail controls • Autoregression: the back section is moved towards the headboard when lifted, reducing applied pressure to the lower back	• Optional panels, side-rails, colors and materials for environmental adaptation • Under-Bed Lighting • Chair position • Concealable controls • Customizable for medical or residential scenarios
Standout functions	• Backup power • Accessory support • Mechanical/Digital head of bed angle indicators • Predefined, programmable positions (cardiac chair, Trendelenburg, etc.) • Usage History (positions, etc.) • Integrated accessories (i.e. active mattress) • Motion Programs	• Lit Controls • Integrated scale • Braden Scale Calculation • Nursing staff reminders • Patient blocking functions • Translation for basic questions • Monitoring (i.e. bed-exit) • Eased CPR control • Motion Support • Treatment/Preventive therapies/systems	• Patient exit alarm • Remote-local alarms • Obstacle detection • Communication functions

Features mentioned in the table (i.e. bed-exit alarms, obstacle detection, advanced motion options, therapy routines, patient and bed history logging, integrated scale, head-of-bed angle monitoring and measurements, patient-blocking, local and remote information on patient conditions, integrated accessory-controls), are all relevant in their own right, but most impactful when controlled under a single patient-care interface. Most of these features are shared among models, and not only isolated cases, representing the increased expectations that their users have developed.

As indicated in section “Changing (and keeping) the face of the medical bed”, design highlights, including patient and caregiver ergonomics and environmental adaptations for clinical and/or residential settings, are most significant and prominently present across the growing family of smart beds. Similarly, access to pre-programmed positions, dedicated accessories and optional features are also largely visible. For instance, 85% of the models highlighted in Table 2 include positions like cardiac chair and automatic CPR (cardiopulmonary resuscitation), and 95% have dedicated accessories like specialty mattresses, IV-holders and options for a fifth castor. From the perspective of robustness and safety, battery backup and eased CPR release, mentioned in Table 3, provide for fast responses to emergencies under power-failure conditions, another functionality mentioned in the current standard.

Supplementing design features, customization of finishings, materials, colors and side-rail styles for a single model are also distinct, but present to a lesser extent (corresponding to a 60% of the models in Table 2), while proving a meaningful addition to the design of dedicated, more patient-conscious environments. Finally, adjustable bed sections (frame width and length), particularly useful for bariatric patients, have become a widespread feature, found in the form of integrated mechanics, or as optional additions, across all models from Table 2.

#### User interfaces

**Fig. 4:**
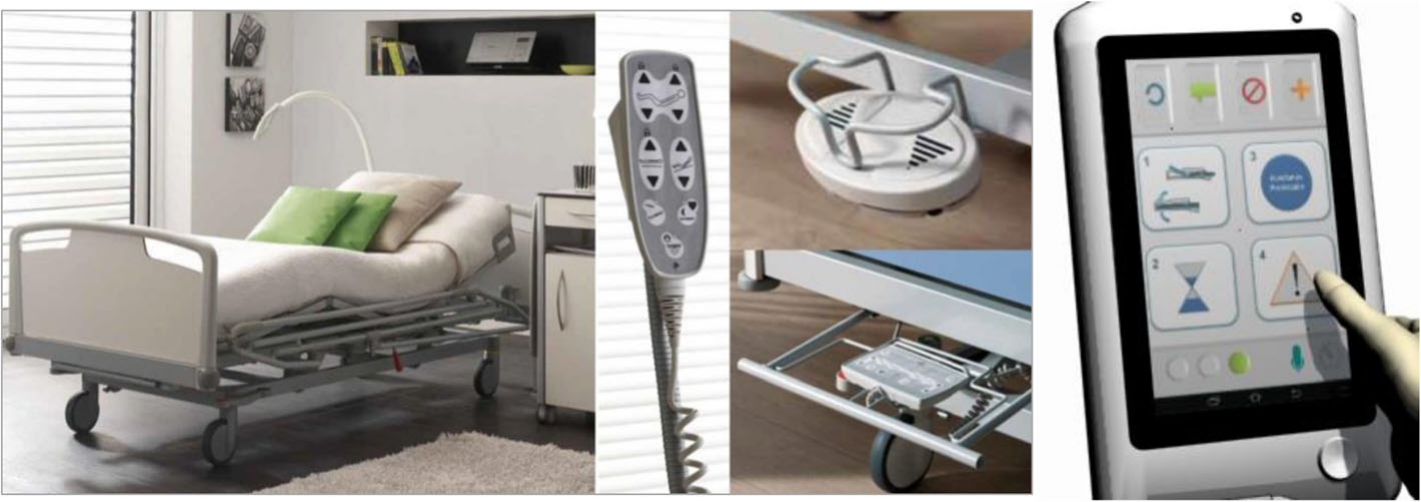
User interfaces in smart beds are multiple, robust and dedicated to the patient and/or caregiver. Integration of new technologies, ergonomics and graphical interfaces allows for improved control over a broader range of functions. Left: Olympia Hospital bed, developed and manufactured by Haelvoet [46]. Right: example of a graphical-user interface designed for the control of a new generation of medical beds [47]. Permission for use of images granted by Haelvoet

As a result of this analysis, it has become evident how user interfaces in medical beds have progressively diversified in this period. Starting with wired hand-held controls (which persist in most models, for instance Joerns UltraCare XT), interfaces have advanced up to solutions with redundant side-rail integrated controls (exterior and interior, i.e. ArjoHuntleigh Enterprise 9000), remote controls, foot-pedals, integrated numeric displays and touchscreens (mostly directed to caregiver control), and variants like hanging controls reachable by the patient (see Linet Eleganza Smart and Haelvoet Olympia). Figure 4 shows examples of these alternatives, supplementing handheld and side-rail controls, which are also visible in Fig. 2. Additionally, current inventions contemplate new integrations between these technologies for future devices [10, 17].

Smart medical-bed interfaces count on the aforementioned, now-standard features like patient blocking, nurse-call functions, networking and interaction with other devices and accessories (i.e. air-pressure mattresses), and redundancy through multiple controls is available for most options, favoring robust and reliable operation. Based on years of interdisciplinary experience, currently-enforced standards have regulated requisites for these interfaces (indicators, size, means of operation) [12]. The addition of advanced graphical means and touchscreens with dedicated user interfaces is currently present in a select number of beds, however (20% of the highlighted cases in this article), while it provides eased access to features like alarm setup and monitoring, and is essential to the control of a growing number of states and functions in smart beds, like bed-history functions. For this reason, their presence is expected to increase in the future.

### Research shaping the future of smart healthcare environments

#### Monitoring and assistive solutions

Available as integrated functions for commercial beds and research proposals, patient monitoring is the most extended group among comprehensive proposals for the care of high-risk or long-term patients. Varied systems for fall [18] and agitation detection, around the bed, can be valuable additions to the medical bed, allowing for patient-aware care environments and active sensor-networks [19]. Similarly, pressure-distribution matrices over the support surface of the bed allow a comprehensive analysis on patient position, and may reduce the risk of developing pressure ulcers [20]. Based on acquired data concerning the state of the patient or device, these monitoring proposals can either show the detected state [21], emit alarms [18], or act autonomously against detected hazards [22, 23]. Patient-motion sensors, in contact with the subject, can perform similar tasks concerning the detection and alert of lack of mobility (Leaf Healthcare Inc. developed one such device), as isolated additions.

Integrated, non-invasive vital-sign acquisition from the bed has also been a matter of research over the past years. A consumer-ready device [24] can be placed under the mattress, and acquire heart and breathing rates, as well as estimate patient motion and register caregiver-response time. Outside the medical bed, entertainment systems with infrared sensors have been valued and are being studied for non-invasive heart rate detection [25].

Head-of-bed angle indication and logging is important for the prevention of secondary conditions associated to immobility, and requisites concerning its state vary depending on the condition of the patient (prevention of pressure ulcers requires low angles, and risk of aspiration in intubated patients requires head-rest angles of over 30 degrees, according to the experience of nursing and medical staff) [26]. While incorporated into high-end current devices, the addition of this functionality to manual devices through MEMS [27] sensors is a low-cost addition with great positive potential [28]. Many current technologies are, likewise, sufficiently developed to allow for low-cost derived solutions, integrating these into less-developed healthcare environments.

Finally, patient-motion assistance is another field of research that is valued for the care of the older and disabled patients, with research solutions [29], as well as consumer-ready devices incorporating such features, so that the device supports patient motion, instead of replacing it.

#### Accessibility interfaces for smart beds

Directed to the treatment of subjects with temporary or permanently-restricted mobility, medical beds benefit from past and current accessibility inventions and developments, both in terms of their actuation-means, as well as in terms of their user-interfaces.

Among these solutions, environmental control units (ECU, see [30] for a review on incipient models of the late 90’s) currently stand out as available additions to the medical bed environment, controlling some medical beds. These systems are among the most used accessibility-technologies by patients with spinal cord injury [31]. Basic inputs to ECUs include accessibility switches, sip-puff controls and pressure sensors (see [32]), and their impact on the quality of life of patients is a matter of research [33]. Over the past decades, this concept has been considered by research solutions for healthcare facilities, with inventions aiming at supplementing bed control [17]. Speech control, for instance, is considered and implemented on a limited number of cases, as an accessory ECU (as the one developed for the Pro Bed Medical Technologies Freedom Bed) or incorporated into some devices [34]. The promise of speech control for these devices is not new, with a patent from 1991 describing an ECU for a hospital bed and its environment [35]. The effectiveness of speech recognition, however, was more limited at that time, as compared to current models and consumer-ready systems.

Another example is the development of Brain-Computer Interfaces [36], powerful technologies for the aid of critically impaired patients, which are seen as possible inputs for ECUs and medical beds with basic actuating functions. This promising technology, however, presents specific difficulties [37] and is under development, limiting its current reach.

#### Technology-assisted healthcare environments

Surrounding the medical bed, the integration of information-technologies into the patient-care environment has changed the way patient-information and treatments are handled. Updated user interfaces, dedicated to patients and caregivers, have emerged over the past decade, both as consumer-ready solutions [38] and research projects [34], covering the management of patient records, and control over the near environment (like TV, lights, etc.). As medical beds become smarter, interaction with these smart environments becomes a possibility [8].

**Fig. 5:**
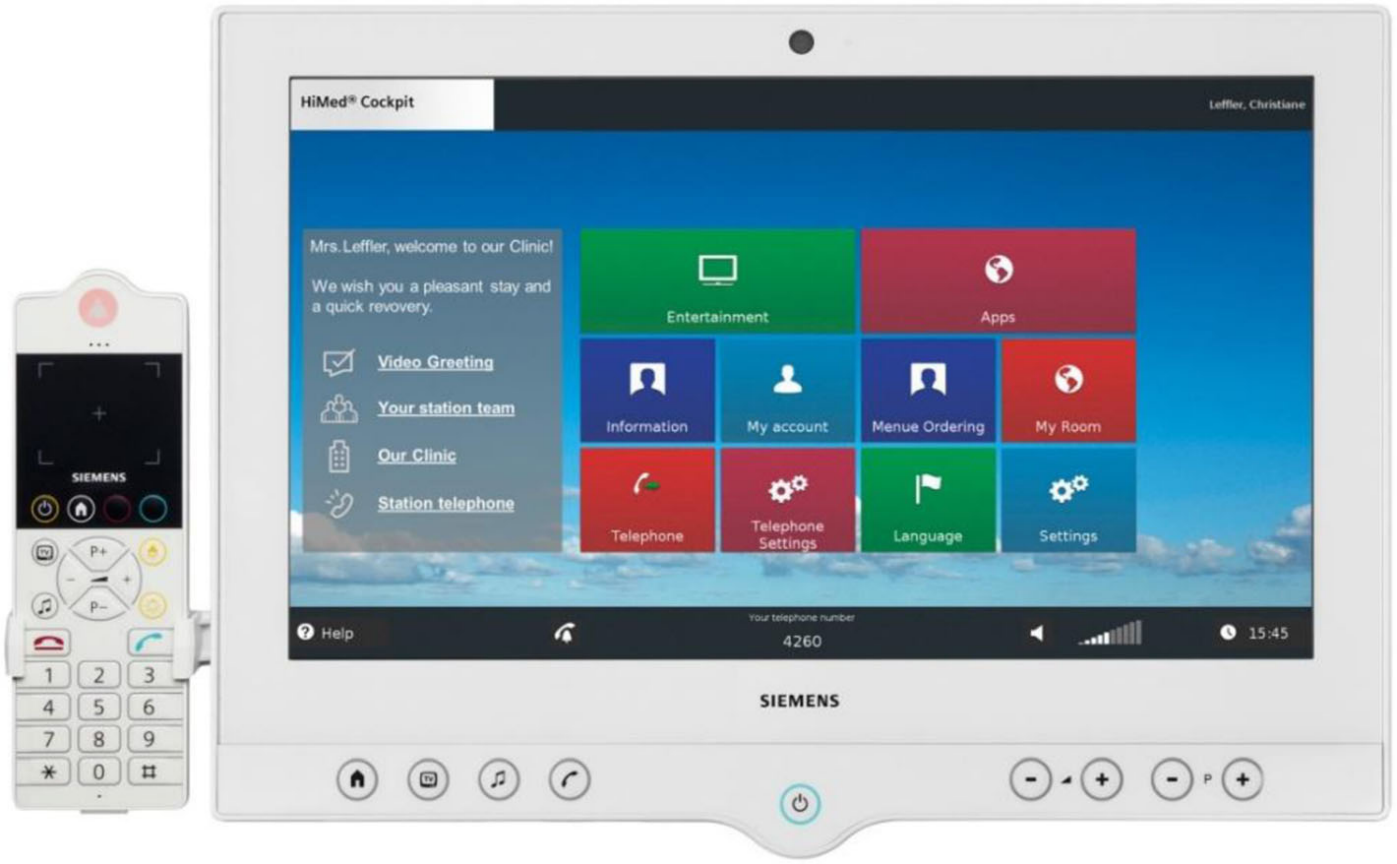
Interactive patient-care systems integrate technology, information and communications into the healthcare environment [38]. Permission for use of images granted by Siemens

A growing trend looks to change the experience of the healthcare environment for patients, providing new means of communication and entertainment at their reach, particularly aimed at patients with restricted mobility (acute, recovering, and long-term patients). These proposals integrate connectivity and a higher control over the environment, while including informative resources (tutorials [38], etc.) concerning different conditions. Even when not including control over the bed itself, these *“*Interactive Patient-Care Systems” [39] may integrate a touchscreen through adjustable stands (e.g. Siemens HiMed Cockpit, visible in Fig. 5).

The development of personalized healthcare and medical devices for injuries and chronic diseases has been deemed as one of the most impactful and feasible challenges to be tackled by biomedical engineers in the near future [40], and the evolution of medical beds and devices in the immediate surroundings of the patient is instrumental to these advances. The core of these projects stems from a thorough vision concerning the patient’s experience of this environment, valuing the possibility of *empowering patients* on their own care, through a more fluid interaction with their surroundings.

## Discussion

Medical beds have changed, in the past decades, from technological, aesthetic, and functional perspectives. Smart medical beds are a comprehensive synthesis of these three: integrated solutions for patient care, assistance and monitoring. Powered by a surge in user technological-awareness, the acceptance of new technologies into smart beds and accessories will likely continue to grow in developed regions, reaching more complex, upgraded, and even bold iterations in the near future.

The future of medical beds will be shaped by the continued, conscious supplement of technologies into the healthcare environment. A prospective analysis on the evolution of healthcare systems will be necessary for the definition of proper strategies, in order to provide better, adapted services (expectations concerning number of beds and resources needed [41]). As for patients and environments, research is heading in the way of providing even further functionality and integration with the medical bed. Embedded monitoring, autonomous responses and accessibility-enabled systems can take full advantage of the potential of these devices, while also posing future challenges in the development of reliable solutions for the severely-disabled.

Technologies implemented into smart medical beds may, at this point, result in derived, low-cost upgrades to other devices, like manually-operated beds, which less-developed regions can benefit from.

While features like autonomy and embedded functionality may hint at an apparent detachment form the work of health specialists at this point in time, the need for multidisciplinary insight will, actually, become more urgent in the development of successful healthcare solutions. Research and study on healthcare-environment related solutions is of great need in a context of a globally-ageing population [42], where disability will have an even greater impact. Accessibility-enabled smart medical beds have the potential of becoming the center of new, comprehensive and patient-conscious healthcare environments.

## Conclusions

Smart medical beds have emerged in the past decades as integrated solutions for patient care, assistance and monitoring, based on a comprehensive, multidisciplinary design process. The global market of medical beds is currently broad, competitive, and still has potential to spread. Dedicated devices for different demographics are developed, and high-end functionality under customizable solutions have become common features, expected of these devices. Research is also continuously promoting novel or updated integrations of technology into this family of devices. It is expected that these changes will continue to spread into further automation and design adaptations, with the smart bed becoming the heart of the smart patient-care environment of the future. The full potential of smart beds will not only be achieved with isolated technological or morphological advances, but when they are seamlessly integrated into the healthcare system, enabling more efficient efforts for caregivers, and more responsive environments for patients.

## Abbreviations

CPR: Cardiopulmonary Resuscitation
ECU: Environmental Control Units
IEC: International Electrotechnical Commission
ISO: International Organization for Standardization
MEMS: Micro-ElectroMechanical System

## Footnotes

1 *Mechatronics* refers to a multi-disciplinary field, combining knowledge from mechanics, electronics, informatics and communications engineering, as well as control theory, into the design of a broad range of products [57], among which are medical devices.
